# Severe Clostridial Pyomyoma following an Abortion Does Not Always Require Surgical Intervention

**DOI:** 10.1155/2011/364641

**Published:** 2011-09-11

**Authors:** Daphna Stroumsa, Eliel Ben-David, Nurith Hiller, Drorith Hochner-Celnikier

**Affiliations:** ^1^Department of Obstetrics and Gynecology, Hadassah-Hebrew University Medical Center, Jerusalem 91240, Israel; ^2^Department of Radiology, Hadassah-Hebrew University Medical Center, Jerusalem 91240, Israel

## Abstract

*Background*. Clostridial infection following pregnancy may be fatal, and surgery is considered as the treatment of choice. We suggest a conservative management in selected cases when preservation of fertility is of major importance. *Case*. A 41-year-old primigravida presented with abdominal pain and fever, one day following dilatation and curettage at 20 weeks of gestation. Her abdomen was diffusely tender, with a uterus enlarged to 20 weeks' gestation. Laboratory studies were consistent with sepsis and hemolysis. CT demonstrated a gas-containing mass compressing the uterine cavity, and presence of air in pelvic veins. Blood cultures were positive for *Clostridium perfringens*. The patient was treated conservatively, with IV antibiotics and fluid resuscitation, and recovered. *Conclusion*. In selected cases of infected myoma complicated by clostridial sepsis, refraining from surgical intervention is a possible therapeutic approach.

## 1. Introduction

The risk of infection following abortion or uterine instrumentation increases in the presence of leiomyomata [1–5]. 

*Clostridium perfringens* (CP) is a ubiquitous gram-positive, anaerobic, spore-forming bacillus. Toxin production by invasive CP infection may lead to fatal clostridial toxic shock syndrome [6, 7]. 

Clostridial uterine infection has high mortality rates [6, 8] and is reported in association with pregnancy, usually following uterine manipulation or instrumentation, cesarean section, abortion, or miscarriage [7, 9].

## 2. Case

**Figure 1:**
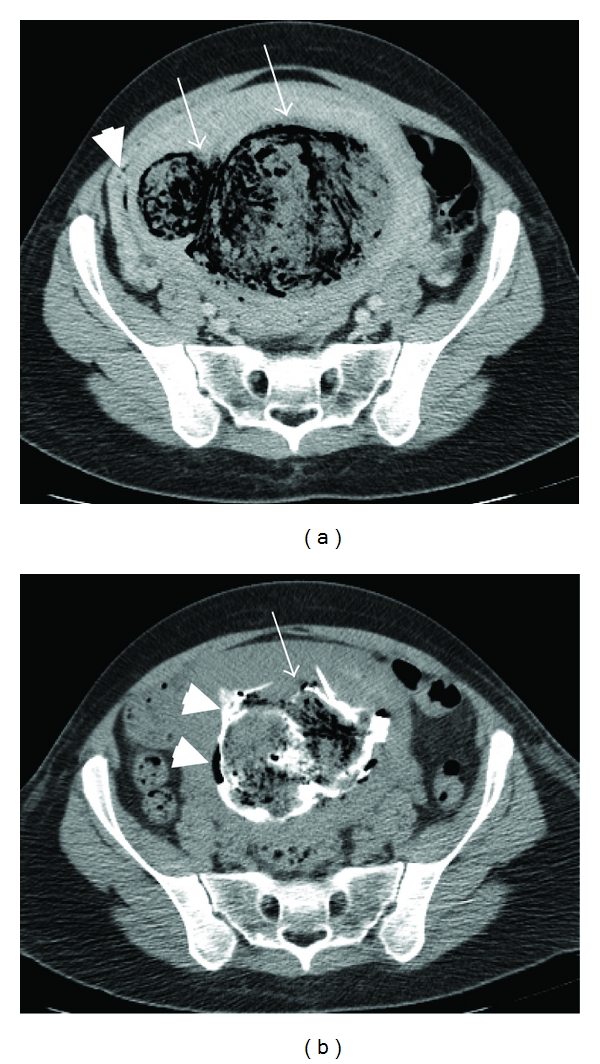
CT of the pelvis demonstrating (a) a large lobular gas containing intramural mass in the left uterine wall (white arrows). The uterine cavity is compressed to the right side (white arrowhead). (b) Following the injection of contrast material into the mass, the contrast is filling the mass (white arrow) and the uterine cavity (white arrowheads).

A 41-year-old primigravida presented with severe diffuse abdominal pain and fever one day following D&C for the termination of the pregnancy at 20 weeks gestation due to trisomy 21. She was known to have a leiomyoma that had been gradually enlarging, from 5 × 6 cm prior to conception to 9.2 × 10 cm in the 11th week of gestation. The patient experienced severe abdominal pain immediately after introduction of the laminariae. Following the surgical evacuation of the uterus, the pain was aggravated, accompanied by a fever of 39°C. Upon admission to our gynecology department, the patient appeared septic, and complained of severe abdominal pain. Her abdomen was diffusely tender, with a uterus enlarged to 20 weeks' gestation. She had a leukocytosis of 20,000 with 94% neutrophils. Abdominal and transvaginal ultrasonography demonstrated a uterine myoma of 11 × 11 cm in the left anterior uterine wall with no evidence of pregnancy residua in the uterine cavity. Enhanced computer tomography (CT) scan of the abdomen and pelvis revealed a 14 × 11 × 10 cm mass in the left uterine wall compressing the uterine cavity (Figure 1(a)), containing gas and heterogeneous debris. Air was also present in the uterine cavity, as well as in the pelvic veins. No air-fluid level, intraperitoneal free air, or fluid was detected. A gas-forming infection of a probably necrotic uterine myoma was suspected.

Broad-spectrum intravenous antibiotic treatment was started. The patient developed hemolysis, with anemia of 6.1 gr/dL. The patient received packed red blood cells and intravenous hydration, with subsequent normalization of her blood count. 

Blood cultures obtained before the initiation of antibiotic treatment demonstrated the presence of *Streptococcus bovis* and *Clostridium perfringens*, sensitive to the antibiotics used.

As abdominal pain and fever persisted, CT-guided aspiration of the infected myoma was attempted. No fluid was aspirated, but contrast material injected into the myoma flowed freely into the uterine cavity and out of the vagina, demonstrating a communication between the myomata and the uterine cavity (Figure 1(b)). 

**Figure 2:**
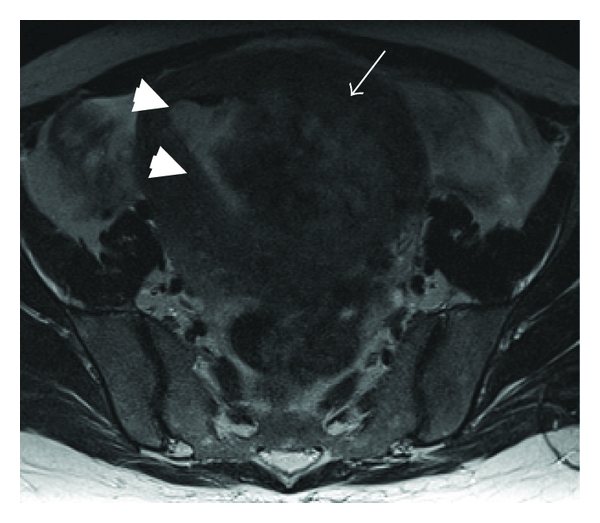
T2-weighted axial MRI image of the pelvis showing the large heterogeneous left intramural uterine mass (white arrow) encroaching into the uterine cavity (white arrowheads).

Magnetic resonance imaging (MRI) preformed on the 13th day of hospitalization revealed the gas containing necrotic intramural mass, 10 × 9 cm in diameter, with an impression of improvement as compared to the CT performed upon admission (Figure 2).

The patient was discharged on the 18th hospitalization day with no evidence of an active infection.

## 3. Discussion

The fulminate course of our patient's illness, with rapid development of sepsis and hemolysis, is typical of clostridial infection. However, our patient remained hemodynamically stable, with no evidence of multiorgan damage. 

 Antibiotic treatment of clostridium infection without debridement is often considered to be inadequate [8]. All 10 cases of pyomyoma complicating pregnancy previously described underwent surgical treatment, with either hysterectomy or myomectomy [1–5, 10–14]. We found no description of a successfully conservative treatment in a similarly severe case in the literature.

Preserving the future fertility of our patient motivated a conservative treatment. The CT exam ruled out perforation of the uterine cavity. Radiographically proven communication between the necrotic mass and the uterine cavity enabled spontaneous drainage of the infection. Furthermore, the immediate contact between the pyomyoma and the uterine cavity would have made conservational surgery difficult or rather impossible.

The standard of care for uterine clostridial infection is surgical. However, in selected cases where fertility preservation is a major consideration, our experience shows that a conservative approach should be considered.
